# State-dependent modulation of thalamocortical oscillations by gamma light flicker with different frequencies, intensities, and duty cycles

**DOI:** 10.3389/fninf.2022.968907

**Published:** 2022-08-23

**Authors:** Kun Wang, Aili Wei, Yu Fu, Tianhui Wang, Xiujie Gao, Bo Fu, Yingwen Zhu, Bo Cui, Mengfu Zhu

**Affiliations:** ^1^Institute of Medical Support Technology, Academy of Military Science of Chinese PLA, Tianjin, China; ^2^Department of Occupational Medicine, Tianjin Institute of Environmental and Operational Medicine, Tianjin, China

**Keywords:** thalamocortical, oscillation, gamma light flicker, entrainment, biophysical model

## Abstract

Rhythmic light flickers have emerged as useful tools to modulate cognition and rescue pathological oscillations related to neurological disorders by entrainment. However, a mechanistic understanding of the entrainment for different brain oscillatory states and light flicker parameters is lacking. To address this issue, we proposed a biophysical neural network model for thalamocortical oscillations (TCOs) and explored the stimulation effects depending on the thalamocortical oscillatory states and stimulation parameters (frequency, intensity, and duty cycle) using the proposed model and electrophysiology experiments. The proposed model generated alpha, beta, and gamma oscillatory states (with main oscillation frequences at 9, 25, and 35 Hz, respectively), which were successfully transmitted from the thalamus to the cortex. By applying light flicker stimulation, we found that the entrainment was state-dependent and it was more prone to induce entrainment if the flicker perturbation frequency was closer to the endogenous oscillatory frequency. In addition, endogenous oscillation would be accelerated, whereas low-frequency oscillatory power would be suppressed by gamma (30–50 Hz) flickers. Notably, the effects of intensity and duty cycle on entrainment were complex; a high intensity of light flicker did not mean high entrainment possibility, and duty cycles below 50% could induce entrainment easier than those above 50%. Further, we observed entrainment discontinuity during gamma flicker stimulations with different frequencies, attributable to the non-linear characteristics of the network oscillations. These results provide support for the experimental design and clinical applications of the modulation of TCOs by gamma (30–50 Hz) light flicker.

## Introduction

The thalamocortical (TC) loop plays a central role in cerebral rhythmogenesis (O’Reilly et al., 2021), and abnormal TC rhythms have been associated with disorders, such as depression, schizophrenia, Parkinson’s disease and Alzheimer’s disease (Niedermeyer, 1997; Llinás et al., 1999; Hughes and Crunelli, 2005; Madadi Asl et al., 2022). Gamma (40 Hz) rhythmic light flicker can entrain cortical gamma neural oscillations non-invasively and externally to restore cognitive dysfunctions or promote learning and memory (Adaikkan et al., 2019; Tian et al., 2021). Understanding the neurocircuit mechanisms of visually evoked entrainment of gamma can be useful when considering the possibility of the therapeutic and clinical adoption of visual gamma stimulation. However, how the stimulation paradigms interact with endogenous neural activity is currently unknown. It is also unclear how the stimulation effects depend on the TC state and stimulation doses, such as light flicker frequency and intensity. Addressing these issues requires a mechanistic understanding and systematic examination of the stimulation effects on TC network dynamics.

Neural oscillations are the rhythmic fluctuations of electrical activity in the central nervous system, which emerge due to the properties of different types of cells and interactions among them (Mathalon and Sohal, 2015). Many biophysical neural networks (NNs) based on mathematical models of different types of cells have been developed to study the characteristics of thalamus or cortex neural oscillations (Li and Cleland, 2013; Li et al., 2017; Chariker et al., 2018; Negahbani et al., 2018; Huang et al., 2021). For example, a unified biophysical thalamic NN model based on the Hodgkin–Huxley formalism was developed to explore the effect of deep brain stimulation (DBS) or repetitive transcranial magnetic stimulation (rTMS) on thalamic neural oscillations (Li et al., 2017). Similarly, a cortical NN model based on the Izhikevich formalism was developed to study the effect of transcranial alternating current stimulation (tACS) on cortical alpha-band (8–13 Hz) oscillations (Negahbani et al., 2018). Later, the abovementioned thalamic and cortical NN models were coupled to study how tACS entrains the endogenous alpha-band oscillations of TC NNs (Huang et al., 2021). These experiment-based models were employed to analyze the interaction mechanism of exogenous electromagnetic stimulation on internal neural oscillation, providing a good reference for this study.

For visually evoked gamma-band (30–100 Hz) oscillations, a neural synchronization may first be generated by retinal mechanisms (Adaikkan and Tsai, 2020). Next, a feedforward network carries the synchronization from the retina to the lateral geniculate nucleus (LGN) and then to the visual cortex (Adaikkan and Tsai, 2020). Thus, information about light flicker frequency is processed at multiple levels along the retinothalamocortical pathway. Early studies have shown the response of retinal ganglion (GC) cells to frequency global flicker stimulation (Schwartz et al., 2007; Schwartz and Berry, 2008) and how to simulate the transmission from the retina to thalamus (Casti et al., 2008; Werner et al., 2008). However, these studies on retinothalamocortical pathway simulation have focused on the transmission of visual information rather than the oscillation effect of networks; a TC oscillation (TCO) NN model in response to light flicker is lacking.

To gain an in-depth understanding of the entrainment mechanism of TCOs induced by gamma light flicker, we developed and investigated a biophysically detailed TCO NN model and explored the stimulation effects depending on the TC oscillatory states and stimulation parameters (frequency, intensity, and duty cycle).

## Materials and methods

### Thalamocortical network structure

We adopted a previously developed thalamic NN model (Li et al., 2017), which was used to study the effect of rhythmic stimulation on neural oscillations, and connected it to a simplified cortex NN model (Susin and Destexhe, 2021), which was used to study the mechanism of the gamma-band (30–100 Hz) oscillation generation.

The thalamic network included 144 relay-model thalamic cells (RTC), 100 reticular inhibitory neurons (RE), 49 high-threshold bursting thalamic cells (HTC), and 64 local interneurons (IN) based on cat physiological data (Hughes et al., 2004; Lörincz et al., 2008). All thalamic neurons were modeled using the Hodgkin–Huxley formalism of point neurons connected with glutamatergic (mediated by both AMPA and NMDA receptors) and GABAergic (mediated by GABAA receptors) synaptic currents (with implemented short-term synaptic depression) or via gap junctions, with the previously described parameter values (Li et al., 2017).

For the cortical part, we reduced the number of neural cells in the original cortex NN model (Susin and Destexhe, 2021) to match the thalamic NN and increased the possibility of a synaptic connection as another cortex NN model that has the same number of cells (Negahbani et al., 2018), including 80 pyramidal (PY) and 20 fast-spiking inhibitory (FS) neurons. All neurons were connected globally with a probability of 0.8, except for the connections within PY neurons, which was 0.5. Individual PY and FS cells were modeled using the Izhikevich formalism for point neurons with the previously described parameter values (Susin and Destexhe, 2021).

**FIGURE 1:**
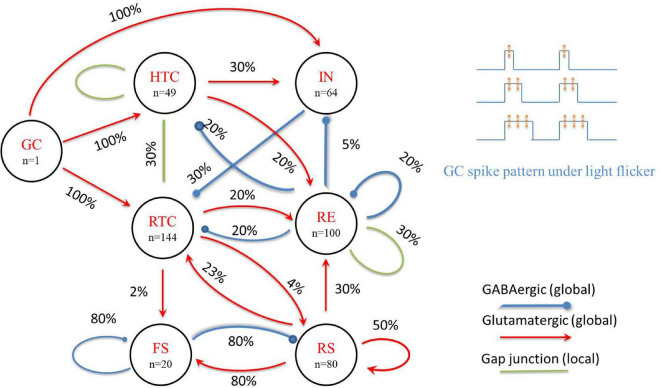
A schematic of the proposed TCO NN model (left) and GC spike pattern under light flicker (upper right). The percentage value represents the possibility of a connection between neuron groups.

The thalamic and cortex NN models were connected in a biologically plausible manner (Izhikevich and Edelman, 2008) to form the TCO NN model (Figure 1); the essential features are highlighted in the following brief description of the model equations.

### Computational model of thalamocortical network

#### Thalamic model

The current balance is described by the Hodgkin–Huxley formalism:


(1) Cmdvdt=-gL(V-EL)-g(V-EKL)KL-∑Iint-∑Isyn

where *Cm* denotes the membrane capacitance, *g*_*L*_ denotes the leakage conductance, *g*_*KL*_ denotes the potassium leak conductance, *E*_*L*_ denotes the leakage reversal potential, *E*_*KL*_ denotes the reversal potential for the potassium leak current (see Supplementary Table 1 for details), and *I^int^* and *I^syn^* denote the intrinsic ionic and synaptic currents, respectively. An ion current is described as follows:


(2) $$Ii=g_{i}m^{p}h^{q}\left(V-E_{i}\right)$$

where *g*_*i*_ denotes its maximal conductance density (see Supplementary Table 2 for details), m denotes its activation variable (with exponent p), h denotes its inactivation variable (with exponent q), and *E*_*i*_ denotes its reversal potential. The gating variable (m or h) kinetic equations satisfy the first-order kinetic model:


(3) $$\frac{dx}{dt}=\phi_{x}\frac{x_{\infty }\left(V,{\left[Ca\right]}_{i}\right)-x}{\tau_{X}\left(V,{\left[Ca\right]}_{i}\right)}$$

where ϕ_*x*_ denotes a temperature-dependent factor, *x*_∞_(*V*, [*Ca*]_*i*_) represents the voltage– or Ca^2+^-dependent steady-state, and τ_*X*_ denotes the voltage– or Ca^2+^-dependent time constant (see Supplementary Table 3 for details). Intracellular calcium is regulated by a simple first-order differential equation of the form:


(4) $$\frac{d{\left[Ca^{2+}\right]}_{i}}{dt}=-\frac{I_{ca}}{zFw}+\frac{{\left[Ca^{2+}\right]}_{rest}-\left[Ca^{2+}\right]}{\tau_{Ca}}$$

where *I*_*ca*_ denotes the summation of all Ca^2+^ currents, w denotes the thickness of the perimembranous “shell” in which calcium can affect membrane properties, z denotes the valence of the Ca^2+^ ion, F represents the Faraday constant, and τ_*Ca*_ denotes the Ca^2+^ removal rate. [*Ca*^2 +^]_*rest*_ is the resting Ca^2+^ concentration (see Supplementary Table 1 for details).

For synaptic currents, the gap junction current is computed as follows:


(5) $$Igap=\left(V_{pre}-V_{post}\right)/R_{g}$$

where *V*_*pre*_ and *V*_*post*_ denote the membrane potentials of the presynaptic and postsynaptic neurons, respectively. *R*_*g*_ is the gap junction resistance (*R*_*g*_ = 100 MΩ for the HTC-HTC synapses, *R*_*g*_ = 300 MΩ for the HTC-RTC and RE-RE synapses), Chemical synaptic currents are calculated as follows:


(6) $$I_{syn}=sDg_{syn}B\left(V\right)\left(V-E_{syn}\right)$$

where *g*_*syn*_ denotes the maximal synaptic conductance, and *E*_*syn*_ denotes the synaptic reversal potential. The function *B(V)* implements the Mg^2+^ block for NMDA currents, the gating variable, s, represents the fraction of open synaptic ion channels and obeys the first-order kinetics:


(7) $$\frac{ds}{dt}=\alpha \left[T\right]\left(1-s\right)-\beta s$$

where [*T*] denotes the concentration of the neurotransmitter in the synapse which is assumed to be a brief pulse that has duration of 0.3 ms and amplitude of 0.5 mM following an action potential in the presynaptic neuron, and α and β denote forward- and backward-binding rates, respectively. Short-term synaptic depression is implemented in all chemical synapses and is modeled by scaling the maximal conductance of a given synaptic channel by a depression variable D, which is expressed as follows:


(8) $$D=1-\left(1-D_{i}\left(1-U\right)\right)\exp\left(-\frac{t-t_{i}}{\tau }\right)$$

where U denotes the fraction of resources used per action potential, τ denotes the time constant of the synaptic vesicle recovery, *D*_*i*_ represents the value of D immediately before the ith presynaptic spike, and *t*_*i*_ represents the timing of the ith spike event. The numerical value of the above parameters are indicated in the Supplementary materials (Supplementary Table 4).

### Cortical neural network model

In summary, the current balance equation is given by


(9) $$Cm\frac{dv}{dt}=-g_{L}\left(v-E_{L}\right)+g_{L}\Delta \exp\left[\frac{\left(v-v_{th}\right)}{\Delta }\right]\tau -w-I_{syn}$$

where *Cm* denotes the membrane capacitance, *g*_*L*_ denotes the leakage conductance, *E*_*L*_ denotes the leaky membrane potential, *v*_*th*_ denotes the effective threshold, and Δ denotes the threshold slope factor. *w* is the adaptation current, given by


(10) $$\tau_{w}\frac{dw}{dt}=a\left(v-E_{L}\right)-w+b\sum_{j}\delta \left(t-t_{j}\right)$$

It increases by an amount b when the neuron emits a spike at *t*_*j*_ and decays exponentially with time scale τ_*w*_. The parameter a indicates the subthreshold adaptation. The synaptic current *I*_*syn*_ is calculated as follows:


(11) $$I_{syn}=g_{E}\left(v-E_{E}\right)+g_{I}\left(v-E_{I}\right)$$

where *E*_*E*_ and *E*_*I*_ denote the reversal potential of excitatory and inhibitory synapses, respectively; *g*_*E*_ and *g*_*I*_ denote the maximal synaptic conductances; they obey the first-order kinetics:


(12) $$\tau_{E,I}\frac{dg_{E,I}}{dt}=-g_{E,I}+Q_{E,I}\sum_{k}\delta \left(t-t_{k}\right)$$

where τ_*E,I*_ denotes the decay time constant. Every time (*t*_*k*_) the presynaptic neuron generates a spike, the excitatory (*g*_*E*_) or inhibitory (*g*_*I*_) synaptic conductance increases by a discrete amount *Q*_*E,I*_ (excitatory or inhibitory synaptic strength, respectively). When *v* = *v*_*th*_, the membrane potential is reset to *v*_*rest*_, which is kept constant until the end of the refractory period. After the refractory period, the equations are being integrated again. The numerical value of the above parameters are provided in the Supplementary Materials (Supplementary Table 5).

#### Formation of oscillations and neuronal heterogeneity

All neurons in the thalamic network received independent Poisson-distributed spike inputs at an average rate of 100 Hz. The random inputs represent both the extrinsic sources of background noise and asynchronous visual input and were exclusively mediated by AMPA receptors modeled as an instantaneous step followed by an exponential decay with a time constant of 5 ms. Alpha, beta, and gamma oscillatory states (with main oscillation frequencies at 9, 25, and 35 Hz, respectively) are formed by changing random input synaptic conductances (2.5, 15, and 25 nS, respectively). Each cortical neuron received an external drive (noise), which was implemented as 80 independent and identically distributed excitatory Poissonian spike trains with a spiking frequency of 2 Hz, as in a previous study (Susin and Destexhe, 2021). To introduce heterogeneity to model neurons, the leakage conductance (*g*_*L*_) of thalamic network neurons is drawn from a uniform distribution within ± 25% of the default value (i.e., 0.0075–0.0125 ms/cm^2^). The leakage conductance variation, random synaptic connectivity, and random external inputs constituted neuronal heterogeneity.

#### Input from ganglion cells

In this study, we focus on the intensity and frequency of global flicker stimulation and set the gamma stimulation frequency increased from 30 to 50 Hz with a 1-Hz step increment. As in a biophysical NN model of the dorsal LGN (dLGN) circuit (Heiberg et al., 2016), the input from GC cells to the TC network is spike trains, which are modeled as a firing-rate based model. Early experimental data showed that GC cells had harmonic firing patterns during flicker sequences, especially the ON-bipolar cells (a type of GC cells) that probably oscillate resonantly at the stimulus frequency (Schwartz et al., 2007; Schwartz and Berry, 2008). Thus, for the entire GC cell network, periodic global flicker stimulation can entrain the resonance response, and the input from GC cells can be modeled as a periodic firing model with a frequency consistent with the flicker stimulation. As the duration of each gamma flicker is very short [about 10 ms, which is close to a GC spike duration (Yan et al., 2016)] and the firing rate of GC cells is positively correlated with the intensity of light stimulation (Einevoll and Heggelund, 2000), the firing pattern of GC cells is simplified to one, two, or three spikes during a flicker to represent different light stimulation intensities. We also assume that the duty cycle is proportional to the number of spikes during each flicker and divide a 40-Hz flicker cycle into six equal parts, considering each flicker can emit up to three spikes of GC cells. Then, the 1/6 duty cycle flicker corresponds to GC cells giving one spike during the 1/6 duty cycle, and the 2/6 duty cycle flicker corresponds to GC cells giving two spikes during the 2/6 duty cycle, and so on (Figure 1, upper right). Instead of using a group of GC cells firing with the flicker frequency, we constructed one GC cell to generate spike trains considering that the flicker is global and connected it to all TC and IN cells consistent with physiological data of cat LGN (Van Horn et al., 2000). The properties of the GC-IN synapse are adapted to give responses in accordance with experimental data, where EPSPs are dependent on AMPA and NMDA activation, and typically, three to four simultaneous synapse activations are required to evoke action potentials in IN cells (Acuna-Goycolea et al., 2008). The response of TCs to GC spike is adapted to experimental data (Blitz and Regehr, 2005), i.e., monosynaptic excitation is assumed, mediated by AMPA receptors with a reversal potential of 10 mV. The synaptic conductance agreed with a retina-LGN transmission model (Casti et al., 2008), where the maximal synaptic conductance of AMPA and NMDA was 0.15 and 0.05 μS, respectively.

#### Stimulation protocol

The computational modeling was implemented using the Brian2 simulator in Python. All simulations were performed using the fourth-order Runge-Kutta [RK(4)] method with a fixed time step of 0.02 ms. After the initial parameters, including the network connection and leakage conductance (*g*_*L*_) of thalamic NNs, were determined, the flicker stimulation of different frequencies (30–50 Hz), intensities (one, two, and three spikes), and duty cycles (1/6–5/6) was applied repeatedly to the same network. The simulation duration of each parameter was 10 s.

### Oscillatory evaluation index

#### Simulated local field potential

Thalamic Simulated local field potential (sLFP) was constructed by filtering the mean membrane potentials across all TCs (Li et al., 2019). Similarly, the cortical sLFP was constructed by filtering the mean membrane potentials of all cortical cells as follows:


(13) $$sLFP=\frac{1}{N}\sum_{i=1}^{N}V^{i},$$

where N denotes the number of cells, and V denotes the membrane potential. The raw sLFP was filtered numerically using a bandpass filter (0.5–80 Hz). The frequency power spectrum of the signal was obtained using the Fast Fourier transform of the filtered sLFP with Python functions firwin. The network oscillation frequency was determined from the position of the spectral peak in the frequency spectrum, and the power spectrum heat map was generated using the Python function heatmap.

#### Synchronization index

The phase of each spike (φ) was computed as follows:


(14) $$\phi =\frac{t_{spike}-t_{lastLFPpeak}}{t_{nextLFPpeak}-t_{lastLFPpeak}}\times 360,$$

where *t*_*spike*_ denotes the spike time, *t*_*last LFPpeak*_ denotes the time of the preceding positive sLFP peak, and *t*_*nextLFPpeak*_ denotes the time of the following positive sLFP peak. Then, the SI was calculated as follows:


(15) $$k=1/N\sqrt{{\left[\sum_{i=1}^{N}\sin\left(\phi i\right)\right]}^{2}+{\left[\sum_{i=1}^{N}\cos\left(\phi i\right)\right]}^{2}},$$

where ϕ*i* denotes the phase of each spike relative to the sLFP peaks and N denotes the total number of spikes for both HTC and RTC cells. The SI measures the degree of mutual synchronization between neurons; when all spikes have identical phases, the SI achieves its maximal value of unity.

#### Oscillation power

The oscillation power is calculated as the (maximal) spectral peak of sLFP. For comparison among different states, the oscillation power was normalized by the original spectral peak (without stimulation).

#### Correlation index

To compute the CI, the peri-event time histogram (PETH) for each of the cells (HTC, RTC, IN, RE, FS, and RS) was generated by dividing the simulation time interval into small bins (2 ms) and summing up the number of spikes in each bin. The CI between two cell groups was determined as the peak of the cross-correlation between the mean-removed PETH of the two cell groups. For the thalamus network, the CI was calculated as the mean of the respective index values for all six pairs of neuronal populations within the thalamic network. For the cortex network, the CI is the same as that of RS–FS. In addition, the CI between the thalamic and cortex networks was calculated as the mean of the respective indices for RS–RTC, RS–RE, and FS–RTC.

#### Entrainment judgment

The network is judged to be entrained, when the flicker stimulation frequency (*f*_*s*_), dominant oscillation frequency (*f*_*d*_), spectral peak power (*P*_max_), and average TC firing rates (FHTC and FRTC) satisfy the following criteria:


(16) $$\left|f_{s}-f_{d}\right|<\in$$

(17) $$\frac{P_{\max}}{P_{0}}>\sigma$$

where *P*_0_ denotes the spectral peak without stimulation; ∈ and σ denote the frequency and amplitude thresholds, respectively (∈ = 1 Hz and σ = 1).

### Electrophysiological recording and flicker stimulation

Four adult male Sprague-Dawley rats weighing about 250 g were used to implement electrophysiological recording. First, the rat was anesthetized by intraabdominal injection with 2% sodium pentobarbital (40 mg/kg), and pain sensitivity was tested by paw pinches. Then, the head of the rat was shaved and immobilized in a standard stereotaxic frame. After a midsagittal incision was made in the scalp, the craniotomy window was carefully made as a 3-mm square hole 4-mm anterior to the Bergman and 4 mm to the right of the midline. Four skull screws were placed in burr holes drilled with a microdrill. Phosphate-buffered saline was used to wash away the bone debris. After the endocranium was stripped, a microwire array (Plexon, 2 × 2, column and row spacing of 250 μm) was implanted in the thalamus. The reference and counter electrodes were connected to the ground bone screws in the skull by silver-coated copper wires. The electrode array was inserted using a micropositioner and advanced about every 40 s at an increment of 100 μm to a depth of 4,000 μm. After a week of recovery, the LFP data were taken by a 64-channel neural acquisition processor (Plexon, Dallas, TX, United States.) and its preamplifier (Plexon, Dallas, TX, United States.). Neural electrophysiological data were filtered by a 4–90-Hz bandpass and analyzed by Neuroexplorer. A 30-W LED light source (SENNO-HSL–39536, Shen Zhen, China) was placed above the rat and triggered by a function signal generator to emit light flicker with different parameters. Data were recorded for 10 min for each flicker stimulation parameter. The flicker was turned on at 5 min.

## Results

### Quantification of the thalamocortical network activity

**FIGURE 2:**
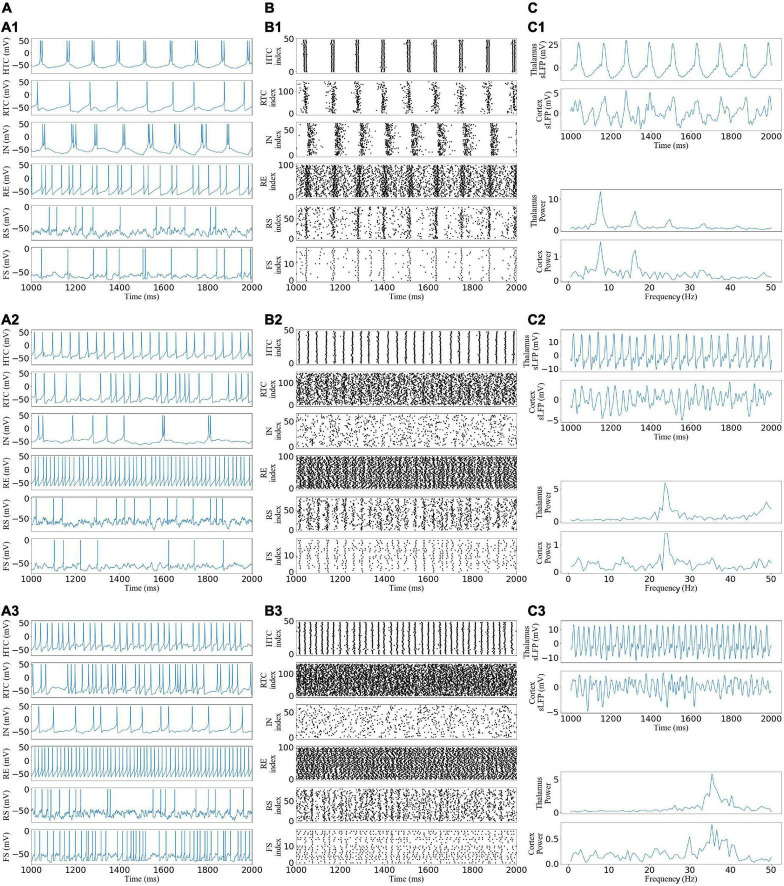
Generation of alpha, beta, and gamma oscillatory states in the TCO NN model under different afferent excitations. **(A)** Voltage traces of representative HTC, IN, RTC, RE, RS, and FS cells each: (A1,A2) alpha, beta, and gamma oscillatory states, respectively. **(B)** Spike rastergrams of HTC, IN, RTC, RE, RS, and FS cells. **(C)** Simulated LFP (top) of thalamus and cortex networks with associated frequency power spectrum (bottom).

From Figure 2, by varying the afferent excitation in the TC cells of the TC network, we generated alpha, beta, and gamma oscillatory states that appear under different behavioral and cognitive conditions in the awake state (Bouyer et al., 1981; Steriade et al., 1993; Brücke et al., 2013). The network reproduces the main features of neural oscillations (Li et al., 2017). For example, as the afferent excitation increased, HTC cells switched from two spike high-threshold bursts (HTBs) during the alpha oscillatory state to one spike tonic spiking during the beta and gamma oscillatory states while remaining well synchronized because of the gap junction connections (Figure 2A1), and the depolarization-induced transition from high-threshold bursting to high-frequency tonic spiking in HTC cells matches the experimental data [Figure 2B in Steriade et al. (1991)]. The thalamus sLFP of the alpha oscillatory state had a strong rhythmic structure (Figure 2B1) and the spectrum revealed the peak power at 9 Hz (Figure 2C1), which was close to the alpha frequency (8.9 ± 1.2 Hz) recorded from freely moving cats during natural wakefulness (Lorincz et al., 2009). The neural oscillations of the thalamic network were transmitted to the cortex network during the three oscillatory states (Figures 2B,C), i.e., the spike rastergrams and simulated LFP of the cortex network had the same oscillatory frequency as those of the thalamus network.

**FIGURE 3:**
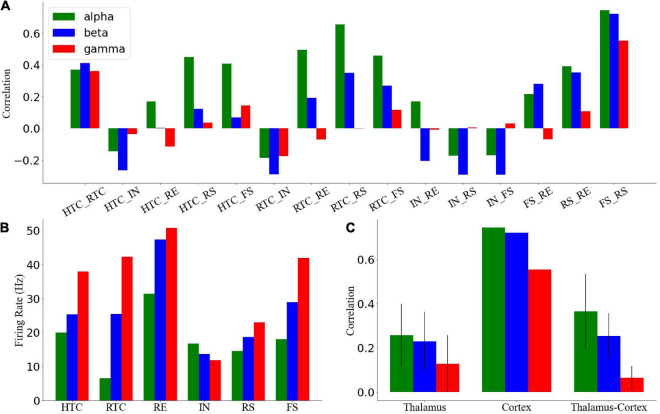
Quantification of network activity. **(A)** Cross-correlation between each pair of the neuronal group during three oscillatory states. **(B)** Average firing rates of all types of neurons across three oscillatory states. Cross-correlation between different groups of thalamic neurons during four oscillatory states. **(C)** Average cross-correlation of the thalamus, cortex, and thalamus to cortex of neuronal groups. Error bars indicate standard deviation.

To quantify the network activity, we calculated cross-CI between different neuronal populations. From Figure 3A, the cross-CI was related to the direct or indirect connection of synapses between two neuron groups, and when two groups of neurons were connected through inhibitory synapses, such as RTC–IN, the cross-CI was negative. When both inhibitory and excitatory synaptic connections existed, such as RTC–RE and HTC–RE, the CI between two groups of neurons gradually became negative with an increase in the oscillation frequency. Further, we calculated the average cross-CIs of the thalamus, cortex, and thalamus to cortex, which decreased with an increase in oscillation frequency (Figures 3B,C), attributable to the change in the RTC cells firing pattern among the three oscillations, which was well synchronized (Figure 2B1) with high CIs for RS and FS cells during the alpha oscillatory state (Figure 3A), but the synchrony fade away for the beta and gamma oscillatory states (Figures 2B2,B3) with low CIs for RS and FS cells (Figure 3A). From the average firing rates given in Figure 3B, we found that the average firing rates of HTC, RTC, FS, and RS cells, which formed the sLFP, increased substantially with an increase in oscillatory frequency; the sLFP power decreased more for the beta and gamma oscillatory states than the alpha oscillatory state (Figure 2C).

Overall, the above results suggest that the thalamic network could generate stable oscillatory states and transmit them to the cortex through RTC cells, so the synchronization of RTC cells would affect the cortex synchronization, which was highly synchronous under the alpha oscillatory state with high cross-CI but poorly synchronous under the beta and gamma oscillatory states.

### Effect of 40-Hz light flicker on signal cells of the thalamocortical network

**FIGURE 4:**
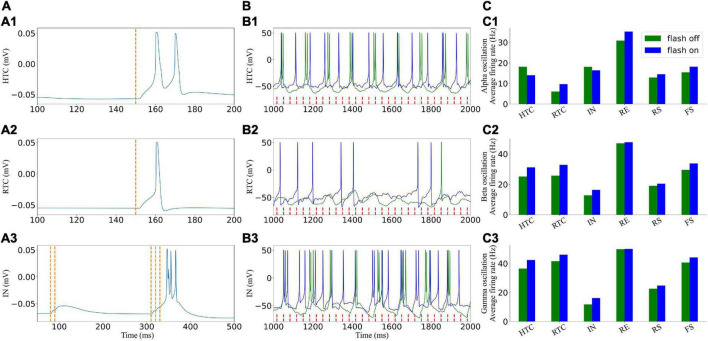
Effects of light (40 Hz) flicker on the TC network signal cells. **(A)** Single cells respond to incoming GC spikes: (A1–A3) HTC, RTC, and IN, respectively. **(B)** Voltage traces of two representative cells in the TC network before (green) and under (blue) light (40 Hz) flicker stimulation. **(C)** Average firing rate when the flicker was on and off: (C1–C3) alpha, beta, and gamma oscillatory states, respectively. The red dotted line represents incoming GC spikes.

Before embarking on the TC network activity under gamma (30–50 Hz) flicker stimulation, we demonstrate the effect of light (take 40 Hz as an example) flicker on signal cells of the TC network (Figure 4). As shown in Figure 4A, the excitatory GC input to the HTC and RTC cells alone would evoke the action potential of the HTC and RTC cells immediately, whereas the IN cells need three GC spikes arriving in a short time to evoke an action potential, which agreed with the experimental data (Blitz and Regehr, 2005; Acuna-Goycolea et al., 2008). To observe the response of cells stimulated by light in the circuit, the response of the TC and IN cells under the alpha oscillation with a low cell firing rate is shown in Figure 4B; not every GC excitatory input could evoke a spike of TC cells in the network in accordance with previous studies (Casti et al., 2008; Heiberg et al., 2016). When the flicker was on, the firing rate of the HTC and IN cells decreased, whereas that of the other cells increased under the alpha oscillation (Figure 4C). Flicker stimulation changed the firing pattern of HTC cells from HTBs to tonic spiking, and the firing pattern of IN cells was a mix of HTBs and tonic spiking under the alpha oscillatory state (Figure 4B).

For the beta and gamma oscillatory states, the HTC cells fired in a tonic spiking pattern originally, and the light flicker increased the firing rate of the TC and IN cells, which further increased the firing rate of other cells in the network. We found that the firing rate of the IN cells decreased with an increase in the network oscillatory frequency ahead (Figure 3B); however, it increased under flicker stimulation, which increased the oscillatory frequency (Figure 4C). The IN cells received excitatory synapses from the GC and HTC cells, inhibitory synapses from the RE cells, and outputed inhibitory synapses to the RTC cells. The firing rate of the RE cells changed slightly (Figures 4C2,C3). Thus, flicker stimulation increased the firing rate of the IN cells by excitatory input from the GC and HTC cells and then enhanced its inhibition on the RTC cells, but this inhibition effect was less than that of light stimulation on the firing rate of the TC cells. Next, we further analyzeed the influence of flicker stimulation on the TC network oscillation.

### Entrainment of 40-Hz light flicker depends on oscillatory states

**FIGURE 5:**
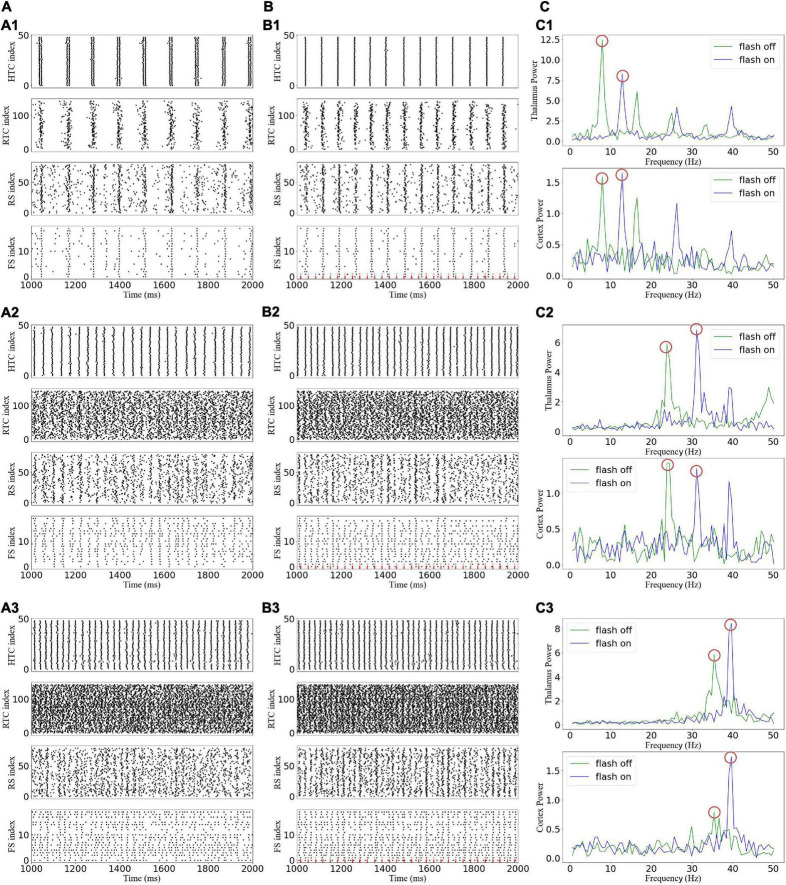
Effects of gamma (40 Hz) flicker on the TC network oscillatory state. **(A)** Spike rastergrams of HTC, RTC, RS, and FS cells before flicker stimulation: (A1–A3) alpha, beta, and gamma oscillatory states, respectively. **(B)** Spike rastergrams of HTC, RTC, RS, and FS cells under flicker stimulation. The red dotted line represents incoming GC spikes. **(C)** Frequency power spectrum of sLFP from the thalamus (top) and cortex (bottom). The red circles indicate the main oscillation frequencies.

A gamma frequency of 40 Hz, which is called the “cortical arousal” or working frequency of the brain, is considered an essential frequency for the interaction of various brain regions (Mcdermott et al., 2018). Experimental results showed that 40-Hz flicker stimulation induced the entrainment of the visual cortex, prefrontal cortex, and hippocampus (Herrmann, 2001; Adaikkan et al., 2019; Jones et al., 2019). Thus, we first explored the effect of the 40-Hz flicker stimulation on TCO (Figure 5). For the alpha oscillatory state, the 40-Hz flicker stimulation switched the firing pattern of the HTC cells and accelerated the oscillation frequency (Figures 5A1,B1), and the sLFP of both the thalamus and cortex revealed the occurrence of 40-Hz frequency oscillation with lower power than the endogenous oscillatory frequency (Figure 5C1). In addition, the 40-Hz flicker stimulation accelerated the endogenous oscillation frequency and induced a 40-Hz oscillation of the beta oscillation state (Figure 5C2). Meanwhile, for the gamma oscillation state, the 40-Hz flicker stimulation changed the endogenous oscillation frequency to 40 Hz and dramatically increased the oscillation power (Figure 5C3), i.e., the 40-Hz flicker stimulation induced a resonant response (Herrmann et al., 2016). Because the cortex oscillation was mainly regulated by the RTC cells, these results suggest that the 40-Hz flicker induced a resonant response of the RTC cells, which made the cortex resonant at 40 Hz.

### Effects of stimulation depend on gamma light frequency and intensity

**FIGURE 6:**
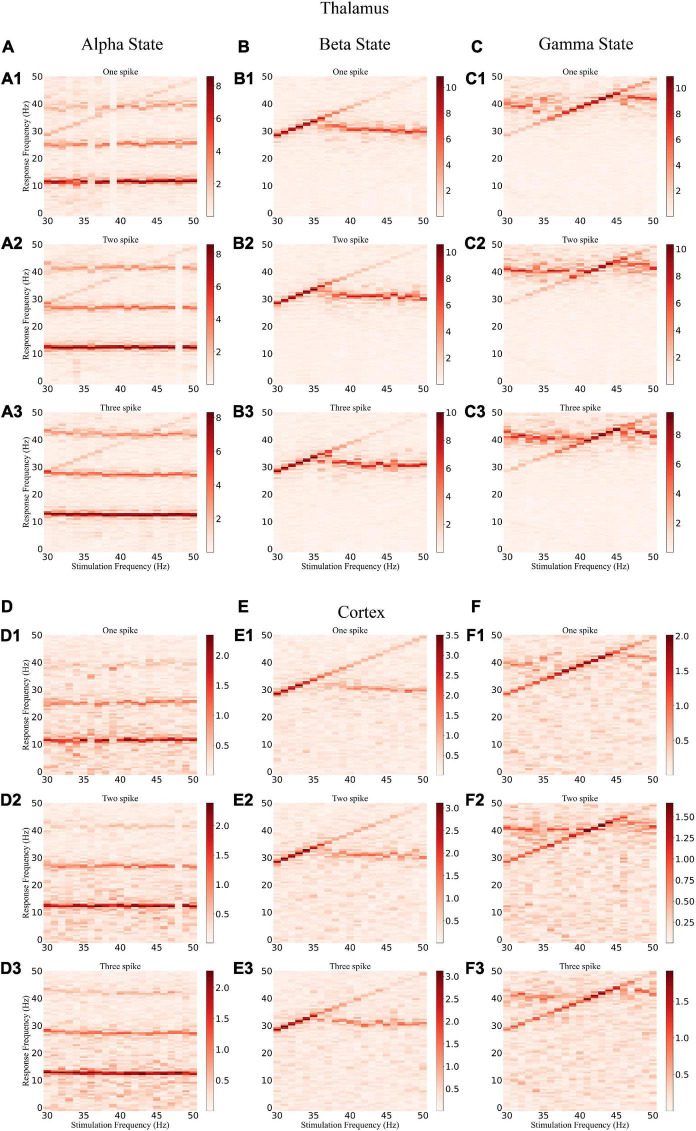
Frequency spectrum heatmap of the sLFP. **(A)** The alpha oscillatory state of the thalamus under light flickers: (A1–A3) intensity of one, two, and three spikes, respectively. **(B,C)** The beta and gamma oscillatory states of the thalamus under light flickers, respectively. **(D–F)** The alpha, beta, and gamma oscillatory states under light flickers of the cortex, respectively.

**FIGURE 7:**
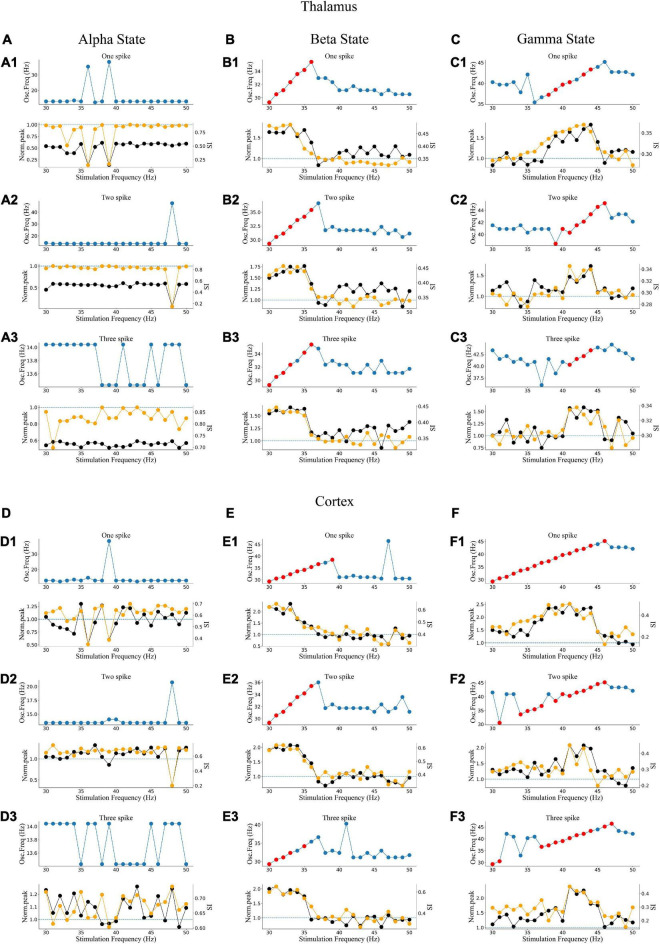
Oscillatory dynamics modulated by gamma flicker. **(A)** Effect of stimulation on the network dynamics of the alpha oscillatory state for the thalamus. Top: Dominant network oscillation frequency; Bottom: Normalized spectral peak (black) along with SI (orange) as a function of stimulation frequency (30–50 Hz) in the presence of three levels of stimulation intensity (A1–A3: one, two, and three spikes, respectively). **(B,C)** The beta and gamma oscillatory states of the thalamus, respectively. **(D–F)** The alpha, beta, and gamma oscillatory states of the cortex, respectively.

To explore how different response patterns unfolded as a function of stimulation frequency and intensity, we generated a frequency spectrum heatmap of the sLFP in response to ascending gamma flicker stimulation (30–50 Hz) with three stimulation intensities for the three oscillatory states (Figure 6). In addition, to examine the oscillation dynamics and network synchronization modulated by gamma (30–50 Hz) flicker, we plot the peak network oscillation frequency, oscillation index (normalized spectral peak) along with the SI as a function of the stimulation frequency in response to the three stimulation intensities for the three oscillatory states in Figure 7.

There were some common effects of the dynamic interaction between stimulation and endogenous network oscillation across the three oscillatory states. First, the flicker stimulations accelerated all three oscillatory states (Figure 6). For the alpha oscillatory state, the endogenous oscillatory frequency changed from 9 to 12 Hz by flicker stimulation (Figures 2C1, 6A,D). For the beta oscillatory state, the endogenous oscillatory frequency changed from 25 Hz to the gamma band (about 30 Hz) by flicker stimulation (Figures 2C2, 6B,E). Meanwhile, for the gamma oscillatory state, the endogenous oscillatory frequency changed from about 35–40 Hz (Figures 2C3, 6C,F). Second, different from the results of electrical or magnetic stimulation (Frohlich and McCormick, 2010; Li et al., 2019), the entrainment frequency range and oscillation power did not increase with the stimulation intensity, suggesting that more inputs at the same stimulation frequency could not yield more entrainment. For example, the entrainment frequency number decreased and the endogenous oscillatory frequency changed slightly with an increase in the stimulation intensity (Figures 7B,C,E,F). Finally, we found that the stimulation with one spike during each flicker induced the largest number of entrainment frequencies in the cortex under a gamma oscillation state (Figure 7F1) and the number of entrainment frequencies decreased non-linearly with an increase in the stimulation intensity (Figure 7F). For example, the entrainment phenomenon disappeared at 30, 32, 33, and 38 Hz of the cortex under the stimulation intensity of two spikes (Figure 7F2) compared with that of one spike (Figure 7F1), and 45-Hz stimulation induced the cortex entrainment for the stimulation intensity of two spikes, which was not entrainment under one spike stimulation intensity. This entrainment discontinuity phenomenon can also be observed in both the thalamus and cortex for the beta and gamma oscillatory states (Figure 7), suggesting that the network has the characteristics of a non-linear system, which agrees with previous studies (Popovych et al., 2005; Li et al., 2019).

Despite the similarities, stimulation also induced substantially different effects among the three oscillatory states. First, under the alpha oscillatory state, it did not evoke entrainment as the flicker frequency was far from the main endogenous oscillatory frequency. Similar to the non-linear characteristics of entrainment, the oscillation disappeared for the 36- and 39-Hz stimulation of one spike intensity and 48-Hz stimulation of two spike intensity (Figure 6A). There were multiple endogenous oscillations (about 14, 28, and 42 Hz) in the range of 0–50 Hz with weakened oscillatory power for high oscillatory frequency under the alpha oscillatory state, explaining the 40-Hz harmonic oscillation occurrence under the 40-Hz flicker stimulation of the alpha oscillatory state (Figure 5C1). Meanwhile, for the beta and gamma oscillatory states, the network was prone to be entrained around the endogenous frequency, which was reflected by the highlighted spectral power along the diagonal in the frequency spectrum heatmap (Figures 6B,C,E,F), and the network synchrony (SI) and oscillation power were enhanced by stimulation during entrainment (Figures 7B,C,E,F). These observations were consistent with previous findings that coupled oscillator systems failed to be entrained by a periodic drive if the stimulation frequency was far away from the average frequency of the system (Antonsen et al., 2008). Second, under the gamma oscillatory state, there were more entrainment frequencies in the cortex than in the thalamus (Figures 7C,F), attributable to the flicker stimulation inducing RTC cell entrainment more easily than both the HTC and RTC cells that made up the thalamus sLFP, and then the entrainment of RTC cells induced cortex entrainment. Finally, gamma (30–50 Hz) flicker stimulation suppressed alpha oscillation in the thalamus, as the normal peak power was always below 1 (Figure 7A), whereas the normal peak power is above 1 when entrainment for beta and gamma oscillations (Figures 7B,C,E,F). In addition, the highest oscillation power did not correspond to the highest SI; 43- or 44-Hz frequency flickers would evoke an entrainment response with the highest oscillation power under the gamma oscillatory state (Figures 7C,F).

### Effects of stimulation depend on gamma (40 Hz) flicker duty cycle

**FIGURE 8:**
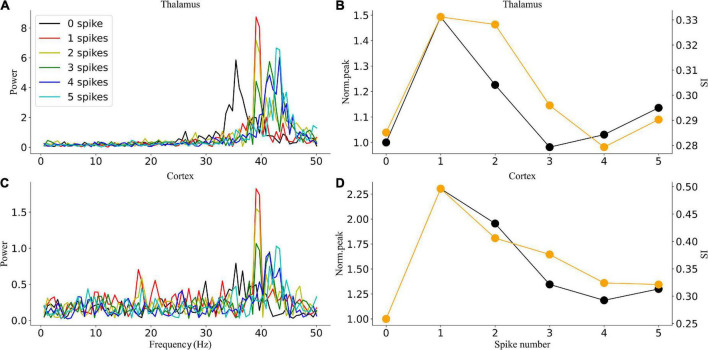
Oscillatory dynamics modulated by 40-Hz flicker with different duty cycles. **(A)** The sLFP spectrum of the thalamus. **(B)** The peak power and SI of the thalamus. **(C)** The sLFP spectrum of the cortex. **(D)** The peak power and SI of the cortex.

A 50% duty cycle flicker stimulation is commonly used in experimental studies to realize gamma oscillation entrainment of the brain (Singer et al., 2018). The effects of light flicker duty cycles have been examined on electroencephalogram responses (Teng et al., 2011); it was found that for the flicker at 11–22 Hz, 50% duty cycles were more reliable than 25 or 10% duty cycles in inducing entrainment in the human occipital lobe. However, whether the duty cycle has similar effects for higher frequency flicker was not addressed. Considering the 40-Hz flicker stimulation as an example, the spectrum, peak power, and SI of the 40-Hz flicker stimulation with different duty cycles under the gamma oscillatory state are shown in Figure 8. Entrainment occurs when the duty cycle is less than or equal to 50%. For a duty cycle above 50%, the endogenous oscillatory frequency exceeds 40 Hz with a decrease in peak power and SI compared to that below 50%. For a duty cycle below or equal to 50%, one spike during each flicker could induce the entrainment with the highest peak power and SI. These results suggest that an effective method to enhance entrainment is ensuring each flicker makes the GC cells give only one spike within a 50% duty cycle instead of increasing the flicker intensity.

### Experimental verification of the simulation entrainment

**FIGURE 9:**
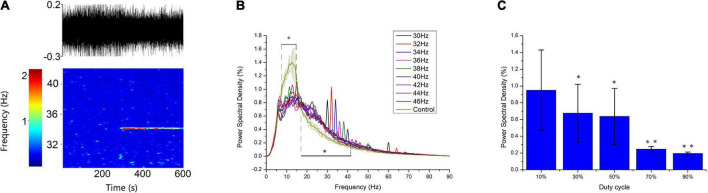
Experimental verification of the simulation entrainment. **(A)** Spectrogram analysis of 34-Hz flicker entrainment. Top: raw LFP of 10 min; Bottom: heatmap of the raw LFP. The flicker was turned on at 300 s. **(B)** A 5-min frequency power spectrum of flicker stimulation with different frequencies on a typic electrode. Error bars indicate s.e.m.; *indicate two-sample *t*-test between the oscillatory power with flicker stimulation and without flicker stimulation for all frequencies within the horizontal line, unequal variance statistical significance, *n* = 9, *p* < 0.01. **(C)** Power spectra density of flicker stimulation with different duty cycles at 40 Hz. Error bars indicate s.e.m.; *indicate two-sample *t*-test between 10% duty cycle and 30% or 50% duty cycle; ^**^indicate two-sample *t*-test between 50% duty cycle and 70 or 90% duty cycle, unequal variance statistical significance, *n* = 16, *p* < 0.01.

To verify the simulation results, we recorded the response of thalamic LFP under different light stimulation conditions. From Figures 9A,B, the entrainment could be observed when the flicker frequency was less than or equal to 40 Hz, and the entrainment of double stimulation frequency occurred at 30 and 32 Hz, attributable to the GC cells having both on and off cells that generated spikes when the light is on and off, respectively, and then formed an oscillation of double stimulation frequency. The spectral peak power was higher at 30, 32, and 34 Hz than at 36, 38, and 40 Hz (Figure 9B), attributable to the endogenous oscillatory power being higher at around 32 Hz than at around 38 Hz. These results suggest that the entrainment was affected by the endogenous oscillation, which corresponds to the oscillatory state. Compared with the frequency power spectrum without flicker stimulation, the low*-*frequency oscillation, such as the alpha oscillation, was suppressed (8–14 Hz), which agrees with the simulation results (Figure 5C1). For all flicker stimulations with different frequencies, the inhibitory effect is significant (Figure 5B). We also found that the oscillatory power was increased for beta oscillation (17–30 Hz). In addition, the flicker stimulation of different duty cycles was tested (Figure 9C); we found that the 10% duty cycle induced entrainment with the highest oscillatory power which agreed with the simulation results (Figure 8), and the oscillatory power was in descending order for duty cycles, 10, 30, 50, 70, and 90% (Figure 9C). Overall, part of the simulation results was verified by the experiments, i.e., the low*-*frequency suppression and oscillatory frequency acceleration effects were statistically significant, and the duty cycle below 50% was easier to induce entrainment.

## Discussion

The TC pathway is the main route of communication between the eye and cerebral cortex (Kremkow and Alonso, 2018). Given how sensitive the human brain is to light, the use of light flickers to modulate the brain is commonly employed to improve cognition and restore cognitive dysfunctions (Martorell et al., 2019; Park et al., 2020; Zheng et al., 2020). In recent studies, visually evoked entrainment was evaluated in mice, revealing that 40-Hz visual stimuli induced 40-Hz entrainment in the visual, somatosensory, and prefrontal cortex, as well as in CA1 (Adaikkan et al., 2019). However, it remains unclear how the stimulation interacts with endogenous neural dynamics. Computational modeling offers a robust tool to examine the impact of rhythmic stimulation on oscillatory brain dynamics, but a brain oscillations network model in response to light flickers is lacking. To close this gap, we developed a TCO NN model and observed state-dependent entrainment of TCOs along with novel response mechanisms, such as entrainment discontinuity. Notably, we observed the modulation of TCOs by flickers from cell dynamics to network responses through a computational model and verified the low-frequency suppression and oscillatory frequency acceleration effects of gamma (30–50 Hz) flicker through experiments. The simulation results offered crucial mechanistic insights into the modulation of TCOs in both dose- and state-dependent manner.

### Entrainment mechanism of gamma (30–50 Hz) flicker

In this study, we investigated the entrainment mechanisms of gamma (30–50 Hz) flicker on TCOs; there were many interesting findings. First, we observed that the TC network carries oscillation information from the retina to the LGN and then to the cortex. Partial GC cells may generate resonant spike trains by light flicker, which agrees with a previous experimental study (Schwartz et al., 2007). In addition, the resonant spike trains induce TC cell entrainment when the stimulation frequency matches the intrinsic frequency of the NN (Figures 6B,C, 7B,C). Then, the entrainment of TC cells further induces the cortex network resonant response (Figures 6E,F, 7E,F). Second, the entrainment is state-dependent. With the HTC cells’ firing pattern changed from a highly synchronous burst mode to a tonic spiking mode, gamma (30–50 Hz) flicker merely accelerated the network oscillation and reduced the oscillation power for the alpha oscillatory state; moreover, all gamma (30–50 Hz) flickers fail to induce entrainment for the endogenous oscillatory frequency, which is almost unchanged by different gamma (30–50 Hz) flicker frequencies and intensities (Figures 6A,D, 7A,D). Meanwhile, for the beta or gamma oscillatory state, entrainment occurs around the endogenous oscillatory frequency with high oscillation power and SI (Figures 7B,C,E,F). Finally, we found that a high firing rate of GC cells induced by high gamma (30–50 Hz) light intensity was not positively correlated with the enhancement of oscillation power or synchronization and the entrainment probability decreased on the contrary (Figure 7). As computational simulation and human EEG studies have shown a positive relationship between stimulus intensity and the level of entrainment (Herrmann et al., 2016; Jones et al., 2019), it may be that the high intensity flicker activated more GC cells to periodic firing which enhance the entrainment. Further investigating the effects of stimulation depending on the gamma (40 Hz) flicker duty cycle, we found that a single periodic input (GC cells give one spike during each flicker) could induce stronger entrainment than a periodic input with more than one spike during each flicker (Figure 8). In addition, part of the simulation results was verified by the experiments. These findings provide valuable insights into the application of gamma (30–50 Hz) flicker stimulation to treat neurological and psychiatric disorders.

### Model limitations

Like any scientific study, there are some limitations to our study. First, the input from GC cells to LGN induced by gamma (30–50 Hz) flicker is simplified, as mentioned above. GC cells had complex harmonic patterns during the flicker sequence; we only simulated the resonant part, whereas some GC cells exhibited period-doubling, period-tripling, or other beat patterns (Schwartz et al., 2007), especially for flickers with frequencies below 34 Hz, as the experimental results show (Figure 9B). For the visual input of the non-resonant part, it may become the noise input and contribute to neuronal heterogeneity. More detailed experiment data are needed to construct realistic oscillation input from the GC cell network to LGN. Second, because the proposed model is a small-scale network focusing on neural oscillation generation and transition, it can only reproduce part of the NN characteristics. The simulation findings provide ideas for the experiments, but further experimental verification of the simulation is required. For example, the dependence of entrainment on the endogenous brain oscillatory states cannot be directly observed by animal experiments in this study. Third, the proposed model is based on data from several animal species, e.g., the GC cells’ responses to flickers were from salamander and mouse, whereas the GC–TC and GC–IN synapse parameters were from cat dLGN. The main features of thalamic physiology seem to be well-conserved across species, but the comparison of model predictions with experimental data from the various species is required.

## Conclusion

In conclusion, we demonstrated that the rhythmic modulation of TCOs by gamma (30–50 Hz) flicker is state-dependent. The endogenous oscillation state of the network determines whether gamma (30–50 Hz) stimulation can induce entrainment. In particular, the gamma (30–50 Hz) flicker induces entrainment under the beta and gamma oscillatory states but not for the alpha oscillatory state. Moreover, it is more prone to induce entrainment for gamma (30–50 Hz) flicker when GC cells give one spike during each flicker compared with multiple spikes. Overall, this study provides insights into how the biophysics of TCOs guides the emergence of complex, state-dependent mechanisms of target engagement, which can be leveraged for the future rational design of novel therapeutic stimulation modalities.

## Data availability statement

The original contributions presented in this study are included in the article/Supplementary material, further inquiries can be directed to the corresponding authors.

## Ethics statement

The animal study was reviewed and approved by Institutional Animal Care and Use Committee (IACUC) of the Chinese Academy of Military Medical Science.

## Author contributions

BC and MZ contributed to the conception and design of the study. KW, AW, and YF contributed to the experiment of the study, analyzed the data, and wrote the first draft of the manuscript. All authors contributed to manuscript revision, read, and approved the submitted version.
