# Supplementary material for: State-dependent modulation of thalamocortical oscillations by gamma light flicker with different frequencies, intensities, and duty cycles
Source: Front Neuroinform. 2022 Aug 23;16:968907. doi: 10.3389/fninf.2022.968907 (PMC9445583; doi:10.3389/fninf.2022.968907)
Supplement: Supplementary file 1 [file Data_Sheet_1.docx]

**S1 Table.** Specific neuron model parameters.

| **Parameter** | **HTC** | **RTC** | **IN** | **RE** |
| --- | --- | --- | --- | --- |
| *C_m_* | 1 μF/cm^2^ | | | |
| *g_L_* | nominal value: 0.01 mS/cm^2^ | | | |
| *g_KL_* | 0 | 0 | 0.02 mS/cm^2^ | 0.01 mS/cm^2^ |
| *E_L_* | -70 mV | | -60 mV | |
| *E_KL_* | -90 mV | | | |
| *Ionic current type contained* | *I*_Na_, *I*_DR_, *I*_H_, *I*_Ca/L_, *I*_Ca/T_, *I*_AHP_, *I*_CAN_, *I*_Ca/HT_ | | *I*_Na_, *I*_DR_, *I*_H_, *I*_AHP_, *I*_CAN_, *I*_Ca/HT_ | *I*_Na_, *I*_DR_, *I*_Ca/T_, *I*_AHP_, *I*_CAN_ |
| *E_Na_* | -50 mV | | | |
| *E_K_* | -90 mV | | | |
| *E_H_* | -43 mV | | | |
| *E_CAN_* | -10 mV | | | |
| *E_Ca_* | $\frac{RT}{2F}log(\frac{{[{Ca}^{2+}]}_{o}}{{[{Ca}^{2+}]}_{i}})$, R=8.31441 J/(mol ^o^K), T=309.15 ^o^K, F=96489 C/mol, ${[{Ca}^{2+}]}_{o}$=2 mM. | | | |
| w | 0.5 $\mu m$ | | | |
| z | 2 | | | |
| F | 96489 C/mol | | | |
| ${[{Ca}^{2+}]}_{rest}$ | 0.5$\mu M$ | | | |
| $\tau$*_Ca_* | 10 ms | | | 100 ms |

**S2 Table.** Maximal conductance densities (mS/cm2) of active ionic currents in the HTC, RTC, IN and RE model cells.

|  | HTC | RTC | IN | RE |
| --- | --- | --- | --- | --- |
| *I*_Na_ | 90 | 90 | 90 | 90 |
| *I*_DR_ | 10 | 10 | 10 | 10 |
| *I*_H_ | 0.01 | 0.01 | 0.05 | – |
| *I*_Ca/L_ | 0.5 | 0.3 | – | – |
| *I*_Ca/T_ | 2.1 | 2.1 | – | 1.3 |
| *I*_Ca/HT_ | 3 | 0.6 | 2.5 | – |
| *I*_AHP_ | 0.3 | 0.1 | 0.2 | 0.2 |
| *I*_CAN_ | 0.5 | 0.6 | 0.1 | 0.2 |

**S3 Table.** Kinetics of gating variables for each channel implemented in the HTC, RTC, IN and RE model cells.

|  | Gating Variable | $\phi_{x}$ | β_x_ or τ_x_ (ms) | α_x_ or *x*_∞_ |
| --- | --- | --- | --- | --- |
| *I*_Na_ | *p=3* | 1 | $\beta_{m}=\frac{-0.28(V-V_{SH}-40)}{1-\exp((V-V_{SH}-40)/5)}$ | $\alpha_{m}=\frac{0.32(V-V_{SH}-13)}{1-\exp(-(V-V_{SH}-13)/4)}$  *V*_SH =_ -30 for HTC and IN  *V*_SH =_ -40 for RTC and RE |
|  | *q=1* | 1 | $\beta_{h}=\frac{4}{1+\exp(-(V-V_{SH}-40)/5)}$ | $\alpha_{h}=0.128\exp(-(V-V_{SH}-17)/18)$ |
| *I*_DR_ | *p=4* | 0.25  (RE: 1) | $\beta_{m}=0.5\exp(-(V-V{}_{SH}-10)/40)$ | $\alpha_{m}=\frac{0.032(V-V_{SH}-15)}{1-\exp(-(V-V_{SH}-15)/5)}$ |
| *I*_H_ | *p=1* | 1 | $\tau_{m}=\frac{1}{\exp(-0.086V-14.59)+\exp(0.0701V-1.87)}$ | $m_{\infty}=\frac{1}{1+\exp((V+75)/5.5)}$ |
| *I*_Ca/T_ | *p=2* | 4.6 | $\tau_{m}=0.4+\frac{0.7}{\exp(-(V+5)/15)+\exp((V+5)/15))}$ | $m_{\infty}=\frac{1}{1+\exp(-(V+62)/6.2)}$ |
|  | *q=1* | 3.7 | $\tau_{h}=300+\frac{100}{\exp(-(V+40)/9.5)+\exp((V+40)/9.5))}$ | $h_{\infty}=\frac{1}{1+\exp((V+86)/4)}$ |
| *I*_Ca/T_  (RE) | *p=2* | 6.9 | $\tau_{m}=0.612+\frac{1}{\exp(-(V+135)/16.7)+\exp((V+19.8)/18.2))}$ | $m_{\infty}=\frac{1}{1+\exp(-(V+55)/7.4)}$ |
|  | *q=1* | 3.7 | When V < -83 mV, $\tau_{h}=\exp((V+470)/66.6)$  When V >= -83 mV, $\tau_{h}=\exp(-(V+25)/10.5)+28$ | $h_{\infty}=\frac{1}{1+\exp((V+83)/5)}$ |
| *I*_Ca/HT_ | *p=2* | 4.6 | $\tau_{m}=0.612+\frac{1}{\exp(-(V+107)/16.7)+\exp((V-8.2)/18.2))}$ | $m_{\infty}=\frac{1}{1+\exp(-(V+34)/6.2)}$ |
|  | *q=1* | 3.7 | When V < -55 mV, $\tau_{h}=\exp((V+442)/66.6)$  When V >= -55 mV, $\tau_{h}=\exp(-(V-3)/10.5)+28$ | $h_{\infty}=\frac{1}{1+\exp((V+58)/4)}$ |
| *I*_Ca/L_ | *p=2* | 4.6 | $\tau_{m}=3+\frac{1}{\exp((V+30)/10)+\exp(-(V+105)/15))}$ | $m_{\infty}=\frac{1}{1+\exp(-(V+10)/4)}$ |
|  | *q=1* | 3.7 | $\tau_{m}=85+\frac{1}{\exp((V+51)/4)+\exp(-(V+410)/50))}$ | $h_{\infty}=\frac{1}{1+\exp((V+25)/2)}$ |
| *I*_CAN_ | *p=1* | 1 | $\tau_{m}=1.6+\frac{2.7}{\exp(-(V+55)/15)+\exp((V+55)/15))}$ | $m_{\infty}=\frac{1}{1+\exp(-(V+43)/5.2)}$ |
| *I*_AHP_ | *p=1* | 1 | $\tau_{m}=\frac{1}{48\cdot[Ca]_{i}^{2}+0.09}$ | $m_{\infty}=\frac{48\cdot[Ca]_{i}^{2}}{48\cdot[Ca]_{i}^{2}+0.09}$ |

**S4 Table.** Parameters for chemical synaptic currents.

| **Parameter** | **HTC-IN** | **TC-RE** | **IN-RTC or RE-TC** | **RE-IN or RE-RE** |
| --- | --- | --- | --- | --- |
| *g_syn_* | 6 nS for AMPA current  3 nS for NMDA current | 4 nS for AMPA current  2 nS for NMDA current | 3 nS for GABA_A_ current | 1 nS for GABA_A_ current |
| *E*_syn_ | 0 | 0 | -80 mV | -70 mV |
| *B(V)* | B(V)=1/(1 + exp(−(V + 25)/12.5) for NMDA current  B(V)=1 for AMPA current | | 1 | 1 |
| $\alpha$ | 0.94 ms^-1^ for AMPA current  1 ms^-1^ for NMDA current | | 10.5 ms^-1^ | |
| $\beta$ | 0.18ms^-1^ for AMPA current  0.0067 ms^-1^ for NMDA current | | 0.166 ms^-1^ | |
| $\tau$ | 700 ms | | | |
| U | 0.07 | | | |

**S5 Table.** Specific cortical NN model parameters.

| **Parameter** | **RS** | **FS** |
| --- | --- | --- |
| *C_m_* | 150 pF | 150 pF |
| *g_L_* | 10 nS | 10 nS |
| *V_th_* | -40 mV | -47.5 mV |
| *E_L_* | -65 mV | -65 mV |
| *Δ* | 2 mV | 0.5 mV |
| *τ_w_* | 500 ms | 500 ms |
| *a* | 4 nS | 0 nS |
| *b* | 20 pA | 0 pA |
| *E_E_* | 0 mV | 0 mV |
| *E_I_* | -80 mV | -80 mV |
| *τ_E_* | 1.5 ms | 1.5 ms |
| *τ_I_* | 7.5 ms | 7.5 ms |
| *Q_E_* | 5 nS | 5 nS |
| *Q_I_* | 3.34 nS | 3.34 nS |
| *V_rest_* | -65 mV | -65 mV |
